# Volatilome: A Novel Tool for Risk Scoring in Ischemic Heart Disease

**DOI:** 10.2174/011573403X304090240705063536

**Published:** 2024-07-08

**Authors:** Basheer Abdullah Marzoog

**Affiliations:** 1 World-Class Research Center «Digital Biodesign and Personalized Healthcare», I.M. Sechenov First Moscow State Medical University (Sechenov University), Moscow, 119991, Russia

**Keywords:** Volatilome, cardiovascular disease, SCORE2, SCORE2-OP, SMART risk score, ischemic heart disease, myocardial infarction

## Abstract

Developing a novel risk score for accurate assessment of cardiovascular disease (CVD) morbidity and mortality is an urgent need in terms of early prevention and diagnosis and, thereafter, management, particularly of ischemic heart disease. The currently used scores for the evaluation of cardiovascular disease based on the classical risk factors suffer from severe limitations, including inaccurate predictive values. Therefore, we suggest adding a novel non-classical risk factor, including the level of specific exhaled volatile organic compounds that are associated with ischemic heart disease, to the SCORE2 and SCORE2-OP algorithms. Adding these non-classical risk factors can be used together with the classical risk factors (gender, smoking, total cholesterol, low-density lipoprotein cholesterol, high-density lipoprotein cholesterol, diabetes mellitus, arterial hypertension, ethnicity, *etc*.) to develop a new algorithm and further program to be used widely.

## 1 INTRODUCTION

Cardiovascular Disease (CVD) remains the leading cause of mortality and morbidity globally [1]. The current scientific focus has transferred from treatment strategies to prevention strategies. The prevention strategies include risk evaluation, such as SCORE2, SCORE2-OP, and SMART risk score [2-5]. These scores use classical risk factors that are not sufficient for accurate determination of the risk of ischemic heart disease. Therefore, we suggest developing a new risk score based on one novel non-classical risk factor. Using the lipidomic biomarkers as a novel pathway for evaluating the risk of development of cardiovascular disease can be of clinical value [6-17]. The currently used scores for assessing cardiovascular disease risk, such as SCORE2 and SCORE2-OP, have some limitations. According to a study, the limitations of SCORE2 include overestimation of risk in some populations, missingness in cohort data, and the need for recalibration in some populations [5]. Another study also mentioned missingness in cohort data as a limitation [18]. However, the new SCORE2 model is based on contemporary data and predicts the risk of incident cardiovascular disease in addition to cardiovascular mortality [19]. A study reports that the SCORE2 algorithm designed for countries with moderate baseline CVD risk was used to categorize the 10-year CVD risk of study participants as low, moderate, high, and very high, and almost all of those with high and very-high risk had low-density lipoprotein cholesterol levels above the treatment thresholds [20]. The current clinical trial suggested the combination of the single-lead electrocardiography using Cardio-Qvark with the exhaled VOCs to diagnose and predict the potential development of IHD in the next 6 months (NCT06181799) [21]. Finally, a study validated and compared the performance of CVD risk prediction models in an Asian population [22].

## 2 NOVEL CARDIOVASCULAR RISK SCORE

Disease prevention is the novel pathway of medical health programs rather than diagnosis and treatment. Therefore, developing a new algorithm for assessing the risk for the development of ischemic heart disease based on the components associated with the exhaled VOCs, additionally to the classically used risk factors in SCORE2 and SCORE2-OP (gender, smoking, total cholesterol, low-density lipoprotein cholesterol, high-density lipoprotein cholesterol, diabetes mellitus, arterial hypertension, ethnicity, *etc*.) to be globally applicable is necessary [15]. The suggested score is more specific for ischemic heart disease and includes the exhaled VOCs as a molecular biomarker of the ongoing changes in the myocardiocytes and coronary artery disease.

An ongoing clinical trial was conducted at Sechenov University by Basheer Marzoog *et al*. to evaluate the effectiveness of the exhaled VOCs as a molecular biomarker for the development of IHD mortality and morbidity events in the next several months and years (NCT06181799). The observed primary results under consideration for publication showed promising results. There are three potential sources of these exhaled VOCs: the first is from the suffering myocardiocytes as a result of lipid peroxidation, the second from the atherosclerotic plaque in the coronary arteries, and the third is from the gut microbiota dysbiosis in individuals with IHD.

## CONCLUSION

A novel principle for the assessment of CVD morbidity and mortality in the next 5-10 years is suggested using the volatilome. In addition to the currently used SCORE2 and SCORE2-OP, volatilome can improve the accuracy and selectivity of the scores for ischemic heart disease.

## AUTHORS’ CONTRIBUTIONS

MB is the writer and researcher, who collected and analyzed the data and revised the manuscript.

## ABBREVIATION

CVD: Cardiovascular Disease
